# Complete Genome Sequence of *Pseudomonas syringae* pv. lapsa Strain ATCC 10859, Isolated from Infected Wheat

**DOI:** 10.1128/genomeA.00024-16

**Published:** 2016-03-03

**Authors:** Jun Kong, Hongshan Jiang, Baiyun Li, Wenjun Zhao, Zhihong Li, Shuifang Zhu

**Affiliations:** a College of Plant Protection, China Agricultural University, Beijing, China; b Institute of Plant Quarantine, Chinese Academy of Inspection and Quarantine, Beijing, China

## Abstract

*Pseudomonas syringae* pv. lapsa is a pathovar of *Pseudomonas syringae* that can infect wheat. The complete genome of *P. syringae* pv. lapsa strain ATCC 10859 contains a 5,918,899-bp circular chromosome with 4,973 coding sequences, 16 rRNAs, 69 tRNAs, and an average GC content of 59.13%. The analysis of this genome revealed several gene clusters that are related to pathogenesis and virulence.

## GENOME ANNOUNCEMENT

*Pseudomonas syringae* is a rod-shaped, polarly-flagellated, Gram-negative, aerobic bacterium, which comprises 64 pathovars with each corresponding to a specific susceptible host species, including tomato, wheat, apple, cocoa, etc. (1). Among these pathovars, *Pseudomonas syringae* pv. lapsa can specifically infect wheat, a widely cultivated food crop, causing yield reduction and economic loss. Though the whole-genome sequence of a model pathogen, i.e., *P. syringae* pv. tomato DC3000, has been published and well studied (2), most of the *P. syringae* strains have their genome sequences unrevealed or uncompleted. To gain a better understanding of the pathogenesis mechanism, as well as the virulence features of *P. syringae*, the whole-genome sequence of the *P. syringae* pv. lapsa, strain ATCC 10859 was determined.

Three paired-end DNA libraries were constructed, with average insert sizes of 380 bp, 490 bp, and 680 bp, respectively, for sequencing with an Illumina MiSeq platform. Meanwhile, a 10-kbp SMARTbell template library was constructed for sequencing in a PacBio RS II single-molecule real-time (SMRT) sequencing platform (3). A total of 4,282,609 (2 × 307-bp paired-end) reads, as well as 19,804 reads with a mean read length of 10,375 bp were obtained, corresponding to sequencing depths above 400× and 30×, respectively. *De novo* assembly of the SMRT reads was performed using the Hierarchical Genome Assembly Process (HGAP) assembly protocol version 3 (4) and the Gepard program (5), which yielded one complete circular chromosome sequence. The Illumina reads were adapter-trimmed using the Skewer program (6) before being used for error correction on the circular sequence. Genome annotations were done by the NCBI Prokaryotic Genome Annotation Pipeline (7). The genome contains a 5,918,899-bp circular chromosome with an average GC content of 59.13%. There are 4,973 coding sequences (CDSs), 16 rRNAs, 63 tRNAs, 1 ncRNA, and 55 pseudogenes.

Many studies have proved that *Pseudomonas* spp. can be used for biocontrol via secrete secondary metabolite (8, 9) and biofilm formation (10, 11). As expected, several secondary metabolism associated gene clusters, such as genes associated with pyrimidine synthesis (ACA40_02470, ACA40_02475, ACA40_10035, ACA40_01095, ACA40_19310, ACA40_20760) and genes associated with benzoate synthesis (ACA40_09900, ACA40_10125, ACA40_24870), were identified in this strain. Meanwhile, 6 fimbrial gene clusters (cup) (ACA40_01475, ACA40_07220, ACA40_09125, ACA40_13630, ACA40_15640, ACA40_22320), which were involved in distinct stages of biofilm formation (12), were identified in this strain. Due to the importance of flagella biosynthesis in the development of biofilm (13), the flagella-associated genes were also checked. In sum, 5 flagellar motor genes (motA:ACA40_02870, ACA40_22280, motB:ACA40_02875, motC:ACA40_16875, motD:ACA40_16870), as well as 4 other flagellar genes (FliY:ACA40_01295,FliL:ACA40_01630, FliK:ACA40_16680, FlhB:ACA40_16685) were found. These results indicate that ATCC 10859 is a potential antagonistic bacterium.

Genome comparison between *P. syringae* pv. lapsa ATCC 10859 and *P. syringae* pv. tomato DC 3000 (2) revealed that about 14.7% (729/4,973) of the CDSs of the first strain cannot find homologs in the latter one. Not including many hypothetical genes annotated in this genome, 43 genes associated with type III secretion system (T3SS) were found. In comparison, there are 82, 64, and 49 T3SS genes annotated with the DC3000, 1448A, and B728a genomes, respectively.

### Nucleotide sequence accession number.

The genome sequence has been deposited at GenBank under the accession number CP013183. Strain ATCC 10859 is available from American Type Culture Collection (ATCC).
